# Excitation-Inhibition Imbalance Leads to Alteration of Neuronal Coherence and Neurovascular Coupling under Acute Stress

**DOI:** 10.1523/JNEUROSCI.1553-20.2020

**Published:** 2020-11-18

**Authors:** Kayoung Han, Myunghee Lee, Hyun-Kyoung Lim, Minwoo Wendy Jang, Jea Kwon, C. Justin Lee, Seong-Gi Kim, Minah Suh

**Affiliations:** ^1^Center for Neuroscience Imaging Research, Institute for Basic Science, Suwon, 16419, Republic of Korea; ^2^Department of Health Sciences and Technology, SAIHST, Sungkyunkwan University, Seoul, 06355, Republic of Korea; ^3^Department of Biomedical Engineering, Sungkyunkwan University, Suwon, 16419, Republic of Korea; ^4^Department of Biological Sciences, Sungkyunkwan University, Suwon, 16419, Republic of Korea; ^5^KU-KIST Graduate School of Converging Science and Technology, Korea University, Seoul 02841, Republic of Korea; ^6^Center for Cognition and Sociality, Institute for Basic Science, Daejeon 34126, Republic of Korea; ^7^Biomedical Institute for Convergence at SKKU, Sungkyunkwan University, Suwon, 16419, Republic of Korea

**Keywords:** acute stress, E/I balance, neuronal coherence, neurovascular coupling, two-photon imaging

## Abstract

A single stressful event can cause morphologic and functional changes in neurons and even malfunction of vascular systems, which can lead to acute stress disorder or post-traumatic stress disorder. However, there is a lack of evidence regarding how acute stress impacts neuronal activity, the concurrent vascular response, and the relationship between these two factors, which is defined as neurovascular coupling. Here, using *in vivo* two-photon imaging, we found that NMDA-evoked calcium transients of excitatory neurons were impaired and that vasodilation of penetrating arterioles was concomitantly disrupted in acutely stressed male mice. Furthermore, acute stress altered the relationship between excitatory neuronal calcium coherence and vascular responses. By measuring NMDA-evoked excitatory and inhibitory neuronal calcium activity in acute brain slices, we confirmed that neuronal coherence both between excitatory neurons and between excitatory and inhibitory neurons was reduced by acute stress but restored by blockade of glucocorticoid receptor signaling. Furthermore, the ratio of sEPSCs to sIPSCs was altered by acute stress, suggesting that the excitation-inhibition balance was disrupted by acute stress. In summary, *in vivo*, *ex vivo*, and whole-cell recording studies demonstrate that acute stress modifies excitatory-inhibitory neuronal coherence, disrupts the excitation-inhibition balance, and causes consequent neurovascular coupling changes, providing critical insights into the neural mechanism of stress-induced disorders.

**SIGNIFICANCE STATEMENT** Acute stress can cause pathologic conditions, such as acute stress disorder and post-traumatic stress disorder, by affecting the functions of neurons and blood vessels. However, investigations into the impacts of acute stress on neurovascular coupling, the tight connection between local neural activity and subsequent blood flow changes, are lacking. Through investigations at the *in vivo*, *ex vivo*, and whole-cell recording levels, we found that acute stress alters the NMDA-evoked vascular response, impairs the function and coherence of excitatory and inhibitory neurons, and disrupts the excitatory and inhibitory balance. These novel findings provide insights into the relevance of the excitatory-inhibitory balance, neuronal coherence, and neurovascular coupling to stress-induced disorders.

## Introduction

Stress is well known to be a critical contributing factor to psychological and neuropathological disorders. In addition to repeated chronic stressors, single acute stressors can produce structural and functional changes in neurons, including neuronal atrophy and disruption of glutamate/GABA transmission (Musazzi et al., 2010; M. Wang et al., 2012) in the hippocampus and mPFC. Acute stress also leads to imbalance between glutamatergic and GABAergic neurons in the amygdala (G. Y. Wang et al., 2016), which has been implicated in anxiety disorders. Moreover, emerging evidence suggests that acute stress induces not only transient neuronal changes but also long-lasting changes in neuroarchitecture. Indeed, a single foot shock significantly alters the apical dendritic length of pyramidal neurons in the mPFC as early as 24 h after stress exposure, and the change lasts for up to 2 weeks (Nava et al., 2015). These findings imply that understanding the structural and functional alterations in neurons that occur following acute stress is an important step in preventing or treating stress-related disorders, such as acute stress disorder and post-traumatic stress disorder.

Stress hormones can impact both neurons and blood vessels and can even affect the neuron-vessel relationship, which is called neurovascular coupling (NVC). NVC is fundamental for brain homeostasis because it enables the supply of appropriate amounts of oxygen and nutrients to activated brain areas. The NVC unit comprises excitatory neurons, inhibitory neurons, glia, and the cerebral vasculature, and the role of each component has been actively investigated (Attwell et al., 2010; Iadecola, 2017). Pyramidal neurons expressing COX-2 increase cerebral blood flow following prostaglandin E_2_ signaling (Lacroix et al., 2015). Cerebral blood flow can also be modulated by GABAergic interneurons, which have bidirectional effects dependent on their expression of vasoactive peptides, such as vasodilatory effects through vasoactive intestinal polypeptide/nitric oxide synthase and vasoconstrictive effects through somatostatin/neuropeptide Y (Cauli et al., 2004; Uhlirova et al., 2016). Given that each neural component is recruited when cerebral blood flow is increased by sensory stimulation (Lecrux et al., 2011), the balance of excitatory and inhibitory neurons (E/I balance) plays an important role in NVC modulation.

A few studies have reported alterations in NVC, such as malfunction of K_ir_ channels in the smooth muscle of arterioles (Longden et al., 2014) and reductions in hyperemia following sensory stimulation (S. Lee et al., 2015), in chronically stressed mice. Moreover, our prior study demonstrated that alteration of the NVC in chronically stressed mice is derived from the impairment of nNOS-expressing GABAergic interneurons, suggesting that the E/I balance is important for NVC homeostasis (Han et al., 2019). However, little is known about whether acute stress also impacts NVC and about the degree of this impact that is dependent on the contribution of each excitatory and inhibitory neuron.

In the present study, we aimed to examine whether acute 30 min restraint stress modulates NMDA-induced neuronal calcium transients and vascular responses by using real-time *in vivo* two-photon imaging. We also used a viral strategy and acute brain slice preparations for simultaneous calcium imaging of excitatory and inhibitory neurons, explored how acute stress affects the neural activities of excitatory and inhibitory neurons and their interactions, and further, investigated whether acute stress affects excitatory and inhibitory transmission and impairs the E/I balance using whole-cell recording. Finally, we examined whether blockade of stress-related corticosterone signaling restores neural activities.

## Materials and Methods

### 

#### 

##### Experimental design and animals

6- or 8-week-old C57BL/6N male mice (OrientBio) and 8-week-old Thy1-GCaMP6f male mice (stock #025393, The Jackson Laboratory) were used. The Thy1-GCaMP6f mice (3 control and 3 stressed mice) were used for *in vivo* two-photon imaging experiments. The C57BL/6N mice were used for the rest of the experiments; specifically, 20 mice (10 control and 10 stressed mice) were used for ELISA experiments to measure plasma corticosterone concentrations, 22 mice (11 slices from 8 control mice, 12 slices from 5 stressed mice, 6 slices from three vehicle-injected mice, 6 slices from 3 RU486-injected mice and 7 slices from 3 spironolactone-injected mice) were used for *ex vivo* two-photon imaging experiments, 13 mice (10 control and 3 stressed mice) were used for immunohistochemistry (IHC) experiments, and 7 mice (3 control and 4 stressed mice) were used for whole-cell recording. All mice were housed in a cage with *ad libitum* access to food and water. The environment was maintained with a 12 h dark/light cycle (light on 9:00 A.M.), a temperature of 24°C–25°C, and 50%–60% humidity. All experimental procedures were approved by the Institutional Animal Care and Use Committee of Sungkyunkwan University.

##### Mouse model of acute restraint stress

Acute stress was induced by movement restraint for 30 min. To avoid circadian rhythm influences, the restraint stress was imposed between 11:30 A.M. and 1:30 P.M. The subjects were immobilized with well-ventilated plastic bags (DecapiCones, Braintree Scientific) in their home cages. During the restraint period, the animals were restricted from food and water intake. The control group mice were allowed to move freely in their individual cages. All experiments were performed immediately after exposure to 30 min of restraint stress.

##### Blood sampling and ELISA

To quantify the plasma concentration of corticosterone, mouse plasma was collected immediately after the 30 min restraint stress period. Mice (control: *n* = 10; stress: *n* = 10) were anesthetized with Zoletil (30 mg/kg, i.p.), and heart blood was collected in heparin-coated tubes (BD Vacutainer, Becton Dickinson). The blood samples were centrifuged at 5000 rpm for 2 × 10 min at 4°C. The concentration of corticosterone in the plasma was measured using a corticosterone ELISA kit (Assaypro) according to the instructions provided in the kit. The absorbance was scanned at a wavelength of 450 nm using a microplate reader (Synergy HT, BioTek). A standard curve was generated using standard solutions, and the sample concentrations were calculated from the standard curve.

##### Virus injection

Six-week-old C57BL/6N mice were anesthetized with 3% isoflurane for induction and 1%-1.5% isoflurane throughout the surgery with an isoflurane vaporizer (VetEquip). The mice were placed in a stereotactic frame on a temperature-controlled heating pad (FHC) to maintain body temperature at 37°C. The skull of each mouse was exposed, and small holes (0.25 mm diameter) were drilled. Virus injection coordinates were 0.9 mm posterior, and ±2.0 and ±3.0 mm lateral from the bregma (in the somatosensory cortex), and the injection depth was 0.6 mm below the pia. To label both excitatory and inhibitory neurons, a mixture of AAV9-hSyn-GCaMP6f (titer: 1.73 × 10^12^ GC/ml) and AAV9-mDlx-mRuby (titer: 7.4 × 10^11^ GC/ml) (produced by the virus facility of the Korea Institute of Science and Technology) was diluted 1:1 in PBS and loaded into a sharp glass pipette (15-20 μm in diameter). A volume of 600 nl per injection site was delivered at a rate of 80 nl/min using a 10 μl Hamilton syringe and syringe pump (Harvard Apparatus). The glass pipette was left in place for 5 min after the injection to minimize backflow. After the virus injections were complete, the drilled holes were covered with dental sponge, the skin was sutured with surgical suture (B. Braun Surgical), and the animals were returned to their home cages. The animals were allowed to recover for at least 2 weeks before the planned experiments were conducted.

##### Brain slice preparation

After 2-3 weeks of virus expression, the mice were decapitated after brief isoflurane anesthesia (3% induction, 2% maintenance). The brains were quickly removed and placed into ice-cold ACSF containing the following: 93 mm NMDG, 2.5 mm KCl, 1.2 mm NaH_2_PO_4_, 30 mm NaHCO_3_, 20 mm HEPES, 25 mm glucose, 5 mm sodium ascorbate, 2 mm thiourea, 3 mm sodium pyruvate, 10 mm MgSO_4_, and 0.5 mm CaCl_2_ that was adjusted to 300-310 mOsm and a pH of 7.4 and bubbled with 95% O_2_ and 5% CO_2_. Coronal slices (300 μm thick) containing the somatosensory cortex were cut with a vibratome (VT 1200S, Leica Biosystems). Then, the slices were incubated in oxygenated ACSF containing the following: 124 mm NaCl, 2.5 mm KCl, 1 mm NaH_2_PO_4_, 24 mm NaHCO_3_, 10 mm glucose, 1 mm sodium ascorbate, 2 mm sodium pyruvate, 2 mm MgSO_4_, and 2 mm CaCl_2_ for 15 min at 34°C. After another 30 min of equilibration at room temperature, the slices were transferred to a recording chamber.

##### Animal surgery to establish a cranial window

Mice (Tg-Thy1-GCaMP6f-GP5.17DKim/J, JAX 025393, The Jackson Laboratory) were initially anesthetized with 2.5% isoflurane in an induction chamber, and anesthesia was maintained with 1%-1.5% isoflurane during surgical procedures. Body temperature was maintained at ∼37°C using a temperature-controlled heating pad. A craniotomy was carefully performed to expose the somatosensory cortex (bregma: −1 to −3 mm, lateral 1-3 mm) using a dental drill (Microtorque II, Ram Products). The dura mater remained intact, and the exposed cortex was hydrated with HEPES-buffered saline (135 mm NaCl, 5 mm KCl, 10 mm HEPES, 10 mm glucose, 2 mm CaCl_2_, 2 mm MgSO_4_) or HEPES-buffered saline-soaked GelFoam sponges (MS0005, Ethicon). After a lack of microbleeding was confirmed, the exposed cortex was covered with a glass coverslip (4 mm, Deckglaser); a partial opening was left at the right side for insertion of a glass micropipette. A metal holding frame was then glued onto the skull to prevent animal head motions during the imaging experiments and to enable adjustment of the angle of the cranial window so that it remained perpendicular to the microscope objective axis. After all the surgical procedures were completed, isoflurane anesthesia was discontinued, and the mice were immediately switched to urethane anesthesia (1.25 g/kg, i.p.) for *in vivo* imaging experiments. Throughout the experiments, the physiological conditions (i.e., heart rate, pO_2_, and respiration rate) were carefully monitored via a paw sensor (PhysioSuite, Kent Scientific) to determine whether they remained within normal ranges. At the end of all experimental procedures, the mice were killed by CO_2_ inhalation in a closed chamber.

##### Two-photon *in vivo* and *ex vivo* imaging

Imaging was performed using a two-photon microscope (TCS SP8MP, Leica Microsystems) equipped with a Ti:Sapphire femtosecond laser source (Chameleon Vison II, Coherent). Imaging was performed using an objective lens (25×, NA 0.95, Leica Microsystems), a green bandpass emission filter (520 ± 50 nm), and a red bandpass emission filter (624 ± 40 nm). Calcium imaging of GCaMP6f signals was conducted in layer 2/3 of the somatosensory cortex on a horizontal plane in the living mouse brain simultaneously with vessel diameter measurement. The cortical vasculature was visualized by retro-orbital injection of Texas Red-conjugated dextran (MW 70 kDa, lysine fixable, 5% diluted in PBS, 1.5 μl/g) ∼10 min before the start of image acquisition. NMDA was focally applied for 50 ms at 6 psi via a pressure-ejection system (Picospritzer II, Parker Hannifin). To avoid the direct pressure and drug effects of NMDA stimulation on the vascular response, we delivered the stimulation at least 150 μm from the target penetrating arterioles in anesthetized mice.

Since it is easier to distinguish the signals of the soma and dendrites of each excitatory or inhibitory neuron in acute brain slices than *in vivo*, acute brain slice calcium imaging was used for further detailed studies. The two-photon laser was excited at wavelengths of 910 nm for measurement of the GCaMP6f signals of all excitatory and inhibitory neurons and 1040 nm for measurement of the mRuby signals of GABAergic neurons to enable identification of interneurons from the GCaMP6f signals of all neurons. Oxygenated ACSF was circulated over the brain slices containing the somatosensory cortex at a rate of 2 ml/min using a peristaltic pump (ISMATEC). To measure the NMDA-evoked calcium responses of both excitatory and inhibitory neurons, a glass pipette (15-20 μm in diameter) filled with 1 mm NMDA solution was positioned 150 μm from the middle of the FOV. NMDA was focally applied for 100 ms at 10 psi via a Picospritzer II. In all *in vivo* and *ex vivo* calcium imaging experiments, images were acquired at 10 Hz and 512 × 512 pixels (1.25× optical zoom, 0.693 μm^2^/pixel).

##### Calcium and vessel image analysis

All imaging data were processed using Fiji (ImageJ) and custom-written code for MATLAB (The MathWorks). The process for calcium data analysis comprised five steps: image alignment, cell ROI selection, separation of excitatory and inhibitory neurons, calcium signal extraction, and further quantitative analysis. First, the image data were aligned using the “Image Stabilizer” plugin in ImageJ (RRID:SCR_002285). Then, the time-series calcium images were preprocessed using a Gaussian filter and the “Subtract Background” plugin to enhance the signal-to-noise ratio. To make masks of activated cell somata, SD images were calculated from images obtained before and after NMDA stimulation and in turn converted into binary images. Clusters with diameters of <5 µm were then removed. Using the “Analyze Particle” plugin, cell masks were constructed from the calcium signals. To assign each mask to an excitatory or inhibitory neuron, inhibitory cell somata were identified from structural images of mRuby signals. Cells with dendritic signals or with no apparent calcium transients were excluded, as were cells directly contacted by the pipette filled with NMDA. Using MATLAB, raw fluorescence traces were extracted for each ROI. The Δ*F*/*F* of each trace was calculated with the equation, (*F* – *F*_0_)/*F*_0,_ where *F*_0_ was defined as the mean fluorescence of the 30 s baseline trace before NMDA stimulation. To determine the properties of individual neurons, the peak amplitude, onset time, time to peak, FWHM, and half-maximum decay time were determined. The onset time was defined as the time at which the fluorescent signal increased in response to stimulation, which was the first time point when Δ*F* was larger than a *z* score of 5; the *z* score was calculated with the equation: (Δ*F* − *F*_0_)/SD of baseline. To determine the coherence of calcium activities, Pearson's or Spearman's correlation coefficient was determined between each pair of calcium traces. To determine the interaction between excitatory and inhibitory neurons, excitatory neurons were rearranged based on the distance from the center of each inhibitory neuron's soma, and the 3 or 5 nearest excitatory neurons were used for further analysis.

In the case of vessel imaging data, the cross-sections of penetrating arterioles were extracted in each trial. To enhance the signal-to-noise ratio, a 2D Gaussian filter with a 1.386 μm FWHM was applied, and image optimization was then conducted using the “Image Stabilizer” plugin in Fiji to correct the movements induced by air-puffed NMDA stimulation. In every image, to quantify an arteriole diameter, intensity profiles were made over entire horizontal or vertical lines, and the FWHM was calculated for each line. Among the calculated values, a maximal value was considered a diameter. This estimation was made for every imaging frame to determine the temporal changes in arteriole diameter. The arteriole diameter changes over time were calculated as Δ*D*(t)/*D*_0_ ×100 (%) = (*D*(t) − *D*_0_)/*D*_0_ ×100 (%), where *D*_0_ and *D*(t) represent the baseline diameter (the average during the 30 s before the NMDA injection) and the diameter at time *t*, respectively.

##### IHC

Mice were perfused through the heart with PBS, pH 7.4, and then with 4% PFA at a rate of 3 ml/min. The brains were extracted, fixed with 4% PFA for 12 h at 4°C, and then placed in a 30% sucrose solution with 0.1% sodium azide solution at 4°C for 3 d. Forty-micrometer-thick frozen coronal sections were prepared using a cryomicrotome (CM 1950, Leica Biosystems) and transferred in 0.1 m PBS. The sections were incubated in −20°C methanol for 10 min, washed in PBS, and incubated in a blocking solution (10% donkey serum in universal blocking solution, 00-8120, Invitrogen) for 1 h at room temperature. Next, the sections were incubated with primary antibodies in PBS overnight at 4°C and washed in PBS for 3 × 5 min. Then, the sections were incubated with secondary antibodies for 2 h at room temperature and washed in PBS for 3 × 5 min. Nuclear counterstaining was performed with 100 ng/ml DAPI solution (1:10,000) in PBS for 10 min. The primary antibodies were rabbit anti-GFP antibodies (1:800, Millipore). Secondary antibodies conjugated with AlexaFluor-488 (1:350, Invitrogen) were used to visualize the signals. Fluorescence images were obtained using a TCS SP8 confocal microscope (Leica Microsystems) and a 20× objective lens. The images were analyzed with ImageJ and Imaris (Bitplane, RRID:SCR_007370) software.

##### Whole-cell recording

Whole-cell recordings of pyramidal neurons in the somatosensory cortex layer 2/3 were acquired in acute coronal brain slices. Borosilicate glass pipettes (BF100-58-10, Sutter Instrument) with resistances ranging from 5 to 8 mΩ were pulled using a laser micropipette puller (PC-10, Narishige). The pipette was filled with an internal solution containing the following: 120 mm Cs-MeSO_4_, 5 mm NaCl, 4 mm CsCl, 10 mm HEPES, 5 mm EGTA, and 5 mm QX-314, pH adjusted to 7.3 with CsOH (278-285 mOsmol). During voltage-clamp experiments, neurons were clamped at either −70 or 0 mV to measure sEPSCs or sIPSCs, respectively, from the same neurons. Whole-cell voltage-clamp recordings were performed using a MultiClamp 700B amplifier (Molecular Devices), filtered at 2 kHz, and digitized at 10 kHz using a Digidata 1550B digitizer (Molecular Devices). Only cells with an access resistance ≤40 mΩ and membrane capacitance ≥35 pF were recorded. Spontaneous events were analyzed with the MiniAnalysis software (Synaptosoft, RRID:SCR_002184) and Clampfit 11 (Molecular Devices, RRID:SCR_011323). For the calculation of E/I ratio of spontaneous events, all the excitatory values were normalized by inhibitory values.

##### Statistical analyses

To determine whether the data were normally distributed, Shapiro–Wilk tests or Kolmogorov–Smirnov tests were performed on the datasets according to sample size. In accordance with the normality outcomes, independent *t* tests or Mann–Whitney *U* tests were used for comparisons of two groups and the Kruskal–Wallis test with Mann–Whitney *post hoc* comparisons or one-way ANOVAs with Bonferroni *post hoc* comparisons were used for multiple comparisons. To determine the correlation coefficient between two variables, Spearman's coefficient (*r*) or Pearson's coefficient (*r*) was calculated depending on the normality results. A value of *p* < 0.05 was considered to be statistical significance for comparisons of two groups, and *p* < 0.0167 (Bonferroni correction) was considered to be significance for multiple comparisons. Statistical analyses were conducted with IBM SPSS statistical software (RRID:SCR_002865).

## Results

### *In vivo* two-photon imaging of the NMDA-evoked vasodynamics of penetrating arterioles and the calcium activity of excitatory neurons in acutely stressed mice

**Figure 1.**
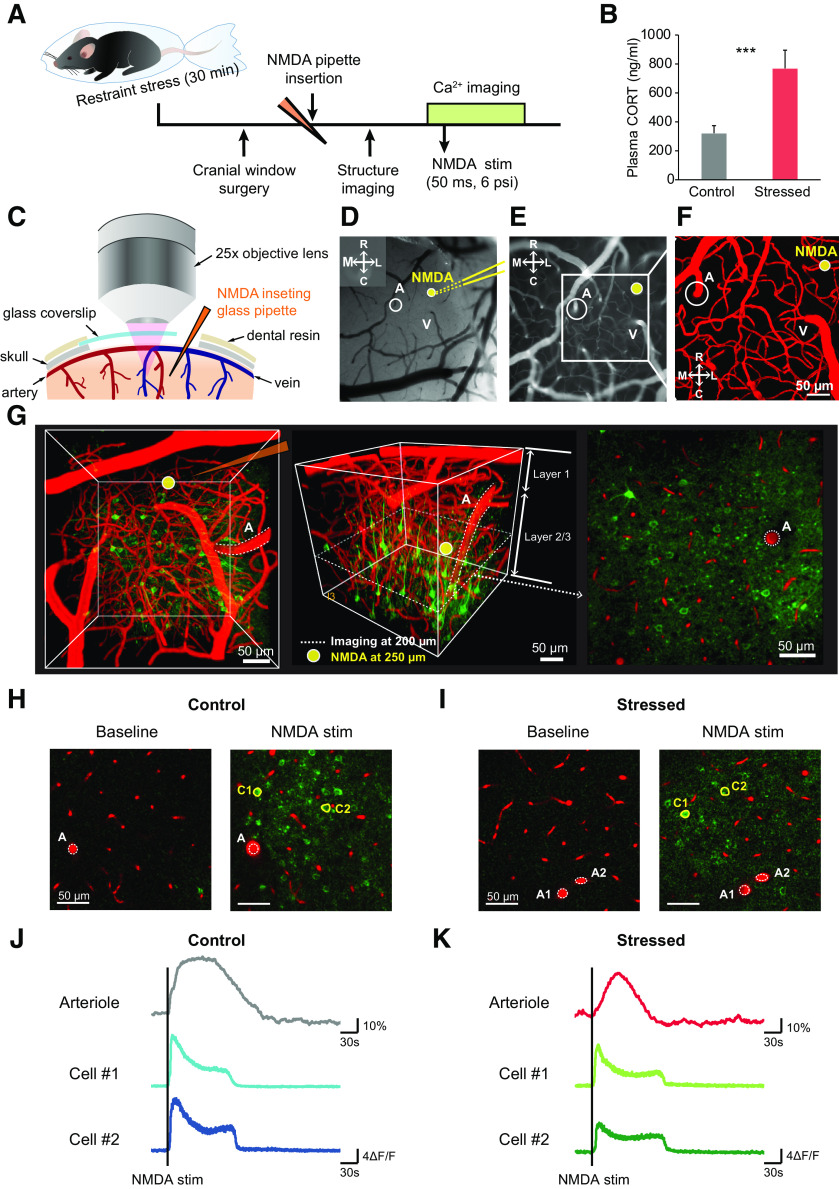
*In vivo* two-photon imaging of the NMDA-evoked excitatory neuronal calcium response and the penetrating arteriolar response. ***A***, Experimental scheme for *in vivo* two-photon calcium and vessel imaging. ***B***, Levels of plasma corticosterone in control and acutely stressed mice (control: *n* = 10; stressed: *n* = 10). ***C***, Schematic images depicting cranial window surgery. In this surgery, a glass coverslip was used to partially cover the window; space was left to allow insertion of a glass pipette during two-photon imaging. ***D–F***, Representative images showing insertion of a glass pipette filled with 1 mm NMDA solution following selection of the ROI. A, Penetrating arteriole; V, vein. Yellow lines indicate the inserted glass pipette. Yellow circle represents the location at which the stimuli were applied. ***G***, Two-photon calcium and vessel imaging was conducted at a depth of 200 μm from the surface, and the stimuli were applied at a depth of 250 μm. ***H***, ***I***, Example fluorescence images of penetrating arteriolar responses and excitatory neuronal calcium responses before (left) and after (right) NMDA stimulation in control and stressed mice. White dashed lines indicate penetrating arterioles before NMDA stimulation. Yellow-masked cells are examples of excitatory cells activated by NMDA. ***J***, ***K***, Example raw trace of penetrating arteriolar changes and calcium transients of yellow-masked excitatory neurons before and after NMDA stimulation. Black vertical lines indicate the points at which the stimuli were applied. ***p < 0.001; independent sampled *t* test.

Stress is a risk factor for sensory processing and pain perception (Khasar et al., 2008; Zheng et al., 2015). Furthermore, several studies have shown that stress causes alterations in glutamate receptor expression (Miyazaki et al., 2012; Toya et al., 2013), synapse formation (Takatsuru et al., 2009), and even the cortical vasculature (S. Lee et al., 2018) in the somatosensory cortex. Thus, we chose to investigate whether a single acute stress can alter the responses of penetrating arterioles and the calcium activity of excitatory neurons in the somatosensory cortex *in vivo*. To induce acute stress, mice were immobilized in plastic bags for 30 min. This well-known acute restraint stress protocol induces several behavioral changes and morphologic and functional alterations in neurons (Rademacher et al., 2008; Roper et al., 2010). The level of corticosterone was higher in the stressed group than in control group (control vs stressed: 322.33 ± 35.54 vs 770.04 ± 89.61 ng/ml, *t*_(11.763)_ = –4.644, *p* < 0.001, independent *t* test; Fig. 1*B*), indicating that the hypothalamic-pituitary-adrenal axis was well stimulated by the acute stress.

NMDA-type glutamate receptors (NMDARs) are the predominant sources of synaptically evoked calcium transients, and the signaling pathway of NMDA-mediated vasodynamics via activation of the enzyme COX-2 has been well established (Lacroix et al., 2015). Thus, we used NMDA stimulation to elicit a local neural network of excitatory neurons and concomitant vascular responses in the somatosensory cortex. After exposure to 30 min of restraint stress, Thy1-GCaMP6f mice were subjected to cranial window surgery for two-photon imaging of calcium activity of pyramidal neurons and penetrating arterioles (Fig. 1*A*). A glass coverslip was used to partially cover the exposed regions of the somatosensory cortex containing penetrating arterioles, and space was left to insert a glass pipette filled with NMDA solution (1 mm diluted in HEPES-buffered saline) (Fig. 1*C–F*). The glass pipette (15-20 μm in diameter) was carefully positioned at a depth of 250 μm, and imaging was performed at a depth of 200 μm to prevent the direct pressure and drug effects of stimulation (Fig. 1*G*). After insertion of the glass pipette, Texas Red-dextran (MW 70 kDa) was injected into the blood to visualize the cortical vasculature. NMDA stimulation triggered penetrating arteriole and calcium transient responses of cortical excitatory neurons in control and acutely stressed mice (Fig. 1*H–K*).

### Acute stress affects the NMDA-evoked pyramidal calcium response, the penetrating arteriolar response, and the relationship between these responses in anesthetized mice

**Figure 2.**
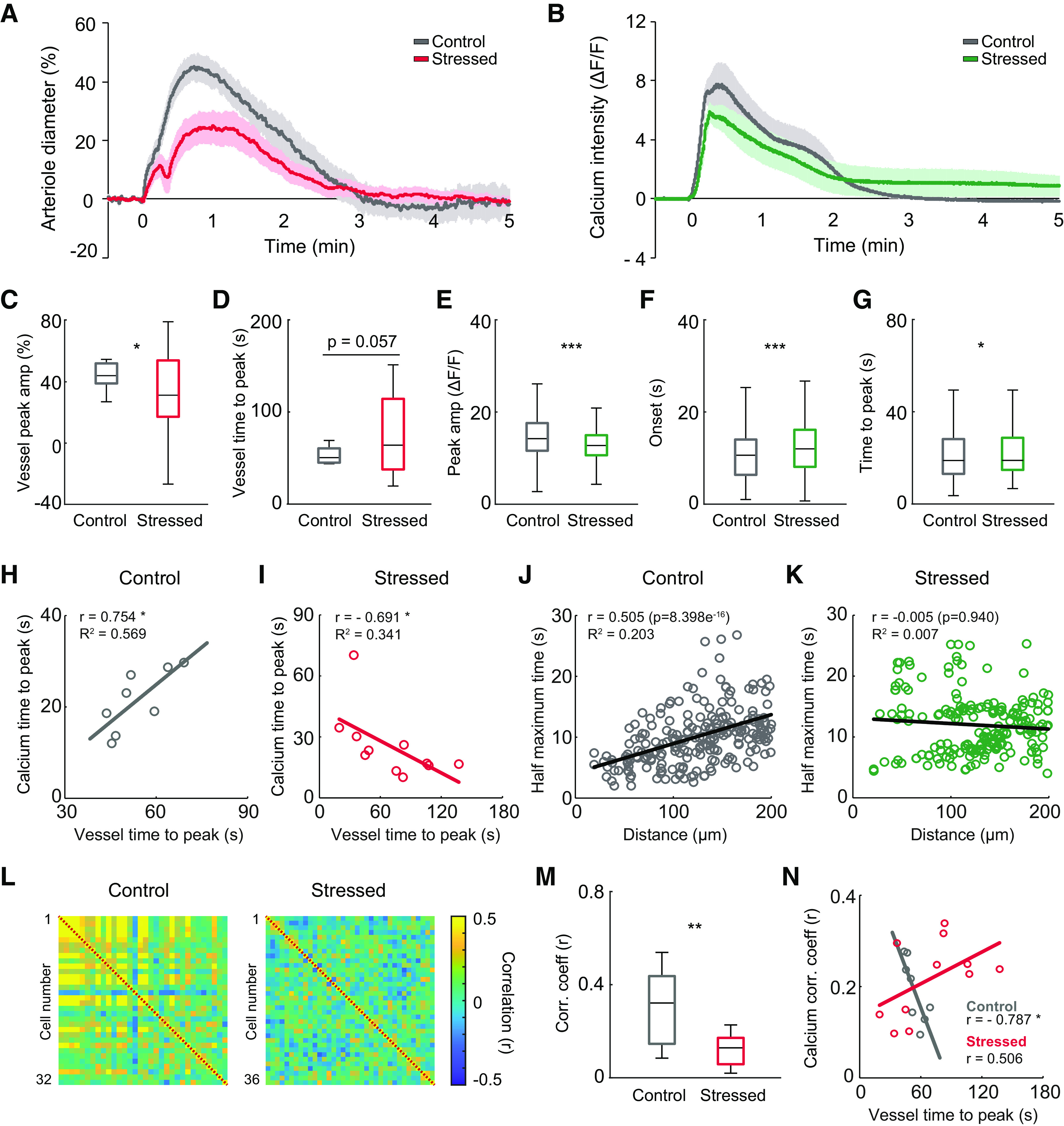
Alterations in NMDA-evoked neuronal calcium responses and penetrating arteriolar responses following acute stress in mice. ***A***, Average vascular traces of penetrating arterioles in response to NMDA stimulation in control and acutely stressed mice. ***B***, Average calcium traces of excitatory neurons in response to NMDA stimulation in control and acutely stressed mice. ***C***, ***D***, Peak amplitudes and times to peak of penetrating arterioles in response to NMDA stimulation in control and stressed mice (control: *n* = 8 vessels from 3 mice; stressed: *n* = 19 vessels from 3 mice). ***E–G***, Peak amplitudes, onset times, and times to peak calcium transient for excitatory neurons in response to NMDA stimulation in control and stressed mice (control: *n* = 456 cells from 3 mice; stressed: *n* = 493 cells from 3 mice). ***H***, ***I***, Scatter plots represent the relationships between time to peak vasodilation and time to peak neural calcium transient in control and stressed mice (control: *n* = 8 trials from 3 mice; stressed: *n* = 11 trials from 3 mice). ***J***, ***K***, Scatter plots represent the relationships between the half-maximum time of neural calcium transients and the distance of the neuron from the site of stimulation. ***L***, Correlation matrices between excitatory neurons during the 2 s following NMDA stimulation for control (left) and stressed mice (right). Right, Colors in scale represent the correlation values. ***M***, Mean correlation coefficients during 2 s of stimulation in control and stressed mice (control: *n* = 8 trials from 3 mice; stressed: *n* = 11 trials from 3 mice). ***N***, Scatter plots represent the relationships between the time to peak of vasodilation and the mean correlation coefficient of evoked calcium transients during the time from calcium onset to peak in control and stressed mice (control: *n* = 8 trials from 3 mice; stressed: *n* = 11 trials from 3 mice). In the box-and-whisker plots, middle line indicates the median, the edges of the box represent the upper and lower quartile values, and the whiskers represent the minimum-maximum range. *p < 0.05; **p < 0.01; ***p < 0.001; Mann–Whitney *U* test.

Stimulation with 1 mm NMDA stimulation (50 ms, 6 psi) elicited vasodilation of penetrating arterioles in both groups, but the peak amplitude of vasodilation was significantly lower in acutely stressed mice than in control mice (control vs stressed: 47.42 ± 4.52 vs 31.69 ± 6.15%, *t*_(24.39)_ = 2.061, *p* = 0.05, independent *t* test; Fig. 2*A*,*C*). Furthermore, the time to peak vasodilation was considerably delayed in stressed mice compared with control mice (control vs stressed: 53.75 ± 3.29 vs 74.53 ± 9.80 s, *t*_(21.58)_ = −2.01, *p* = 0.057, independent *t* test; Fig. 2*D*), indicating that the single acute stressor altered the NMDA-evoked vasodynamics of penetrating arterioles.

Similarly, the peak amplitudes of NMDA-evoked calcium transients from excitatory neurons were notably lower in acutely stressed mice than in controls (control vs stressed: 15.00 ± 0.25 vs 12.95 ± 0.19 ΔF/F, *U*_(456,493)_ = 86,175, *p* < 0.001, Mann–Whitney *U* test; Fig. 2*B*,*E*). Moreover, the onset time and time to peak calcium transient were considerably longer in acutely stressed mice than in controls (onset: control vs stressed: 11.30 ± 0.43 vs 12.55 ± 0.29 s, *U*_(456,493)_ = 96,454.5, *p* < 0.001; time to peak: control vs stressed: 21.85 ± 0.58 vs 27.27 ± 1.27 s, *U*_(456,493)_ = 103,998.5, *p* = 0.046, Mann–Whitney *U* test; Fig. 2*F*,*G*).

After confirming the alterations in vascular responses and calcium transients following NMDA stimulation in acutely stressed mice, we examined whether the NVC relationship was altered by determining the correlation between evoked calcium transients and evoked vascular responses. A shorter time to peak calcium transient was associated with a shorter time to peak vasodilation in control mice; the times to peak vascular and calcium response were significantly positively correlated in these mice (*r* = 0.754, *p* = 0.031, Spearman's coefficient; Fig. 2*H*). In contrast, a negative correlation between these times was observed in acutely stressed mice (*r* = −0.691, *p* = 0.023, Spearman's coefficient; Fig. 2*I*), showing that NVC dysfunction occurred under acute stress.

To determine the cellular signal propagation derived by the NMDA stimulus, the relationship between the time to half-maximum and the distance from the stimulus site was determined. In control mice, a greater distance between the excitatory neuron and the stimulus was associated with a longer time to half-maximum; these variables were significantly positively correlated (*r* = 0.505, *p* < 0.001, Pearson's coefficient; Fig. 2*J*). However, the positive correlation was completely abolished in acutely stressed mice (*r* = –0.005, *p* = 0.94, Pearson's coefficient; Fig. 2*K*), implying that propagation of the cellular signal derived from the NMDA stimulus was impaired by acute stress.

The NVC dysfunction that occurs during acute stress may be because of ineffective neural coherence. To directly assess the strengths of neural networks of excitatory neurons, the correlation coefficients between initial 2 s calcium traces of excitatory neurons were calculated (Fig. 2*L*). When the correlation map was compared between groups, the correlation was found to be considerably weaker in acutely stressed mice than in control mice (control vs stressed: 0.30 ± 0.06 vs 0.12 ± 0.02, *t*_(8.621)_ = 2.95, *p* = 0.017, independent *t* test; Fig. 2*M*). Since we observed impaired neuronal coherence of excitatory neurons in stressed mice, we explored whether the excitatory neural correlation was related to the vascular response. In controls, a higher calcium correlation coefficient was associated with a shorter time to peak vasodilation (*r* = –0.787, *p* = 0.02, Spearman's coefficient), but this relationship did not exist in stressed mice (*r* = 0.506, *p* = 0.112, Spearman's coefficient; Fig. 2*N*). These findings suggest that neuronal coherence is highly associated with the penetrating arteriole vascular response.

### Verification of viral transduction for simultaneous calcium imaging of excitatory and inhibitory neurons in the somatosensory cortex

**Figure 3.**
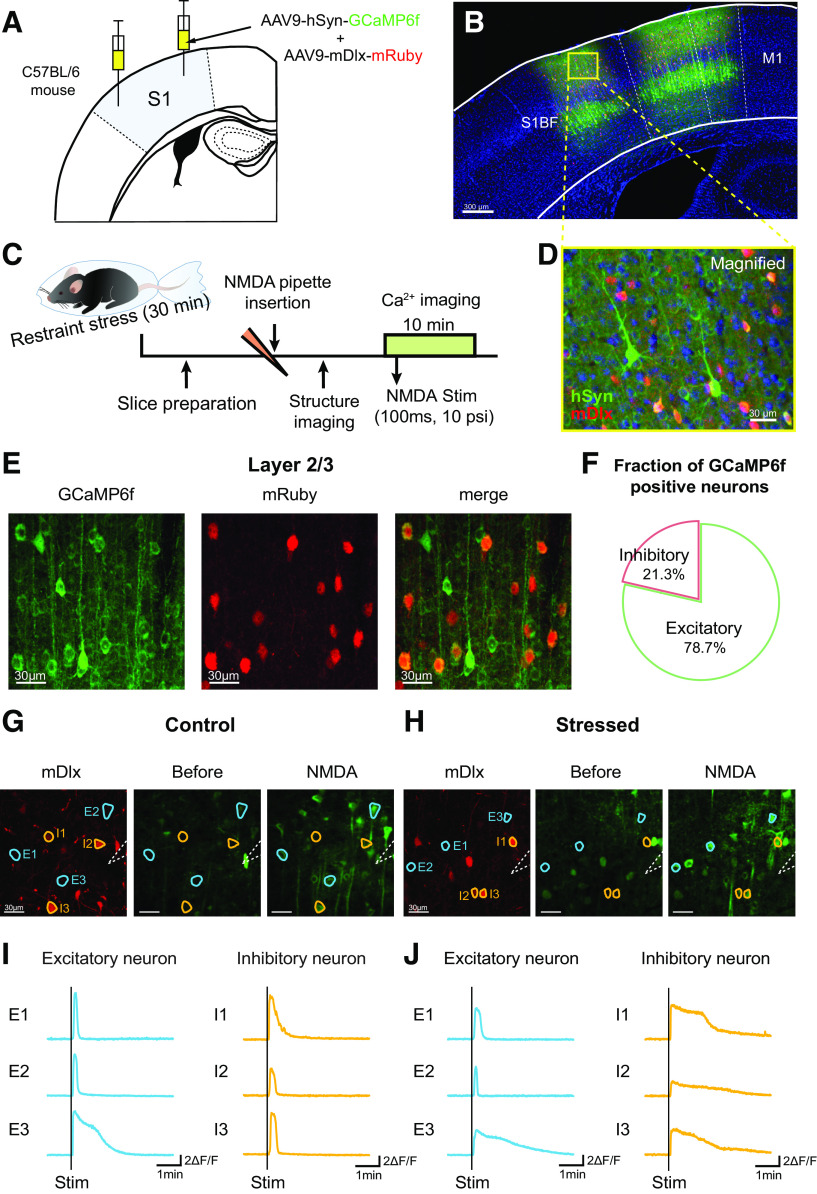
*Ex vivo* two-photon calcium imaging of the concurrent calcium transients of excitatory and inhibitory neurons elicited by NMDA stimulation. ***A***, Schematic image showing the protocol for virus injection. A mixture of AAV9-hSyn-GCaMP6f and AAV9-mDlx-mRuby was injected into the primary somatosensory cortex. ***B***, ***D***, Confocal images represent the expression of the injected viral constructs (green represents GCaMP6f; red represents mRuby; blue represents DAPI) in the primary somatosensory cortex. ***C***, Experimental scheme for *ex vivo* calcium imaging. Two to 3 weeks after virus injection, the mice were immobilized with plastic bags for 30 min, and then *ex vivo* calcium imaging was conducted. ***E***, Representative images showing all neurons (labeled with GCaMP6f; left) and inhibitory neurons (labeled with mRuby; middle) in layer 2/3 of the somatosensory cortex and a merged image (right). In the merged image, the yellow cells are inhibitory neurons expressing GCaMP6f. ***F***, Fraction of GCaMP6f-positive neurons that were positive or negative for the mDlx enhancer, a marker of inhibitory neurons, in layer 2/3 of the somatosensory cortex; ∼21.27 ± 2.20% of GCaMP6f-positive neurons were inhibitory neurons (in a total of 25 sections from 7 mice). ***G***, ***H***, Example fluorescence image of mRuby-positive inhibitory neurons imaged at 1040 nm (left). Representative calcium images showing the SD of neural activity (GCaMP6f) before (middle) and after (right) NMDA stimulation in control and stressed mice. White dashed lines indicate the glass pipette filled with 1 mm NMDA solution. Blue-masked cells are representative excitatory cells activated by NMDA. Yellow-masked cells are representative inhibitory cells activated by NMDA. Scale bar, 30 μm. ***I***, ***J***, Example raw calcium transients of blue-masked excitatory (blue) and yellow-masked inhibitory cells (yellow) before and after NMDA stimulation. Black vertical lines indicate the points at which the stimuli were applied. Calibration: vertical, 2 ΔF/F; horizontal, 1 min.

Functional excitatory neuronal coherence is closely related to the vascular response. Since excitatory and inhibitory neurons precisely regulate cortical neuronal networks, we next sought to determine whether acute stress affects not only excitatory neurons but also inhibitory neurons and to elucidate how each neuron type contributes to neuronal networks. To perform simultaneous calcium imaging of excitatory and inhibitory neurons, we injected a 1:1 mixture of AAV9-hSyn-GCaMP6f and AAV9-mDlx-mRuby into the somatosensory cortex (Fig. 3*A*,*B*). Use of the pan-neuronal promoter synapsin (Syn) induces expression of GCaMP6f, a genetically encoded Ca^2+^ indicator, in all neurons. Additionally, the mDlx enhancer, which is known to specifically target GABAergic interneurons (Dimidschstein et al., 2016), was used to distinguish excitatory and inhibitory neurons. This strategy ensured that only cortical inhibitory neurons coexpressed GCaMP6f and mRuby (Fig. 3*D*). Our histology results showed that inhibitory neurons accounted for 21.27 ± 2.20% of the GCaMP6f-positive neurons in somatosensory cortex layer 2/3 (Fig. 3*E*,*F*). These results are consistent with previous studies reporting that GABAergic interneurons in the cortex account for ∼10%–20% of all neurons (Rudy et al., 2011).

### Deficits in NMDA-evoked excitatory and inhibitory neuronal calcium transients in acute brain slices under acute stress conditions

To examine the effects of acute stress on excitatory and inhibitory neuronal activities, we measured the NMDA stimulation-evoked calcium activity of both types of cortical neurons in acute brain slices. Before calcium imaging was performed, mRuby structural images were acquired at 1040 nm to enable isolation of the calcium activity of inhibitory neurons from all GCaMP6f signals (Fig. 3*G*,*H*, left). Calcium imaging of GCaMP6f signals in response to 1 mm NMDA stimulation (100 ms, 10 psi) was performed at 10 Hz for 10 min (Fig. 3*G*,*H*, middle, right). Only activated neurons coexpressing mDlx and GCaMP6f were regarded as inhibitory neurons; the rest were regarded as excitatory neurons (Fig. 3*I*,*J*).

**Figure 4.**
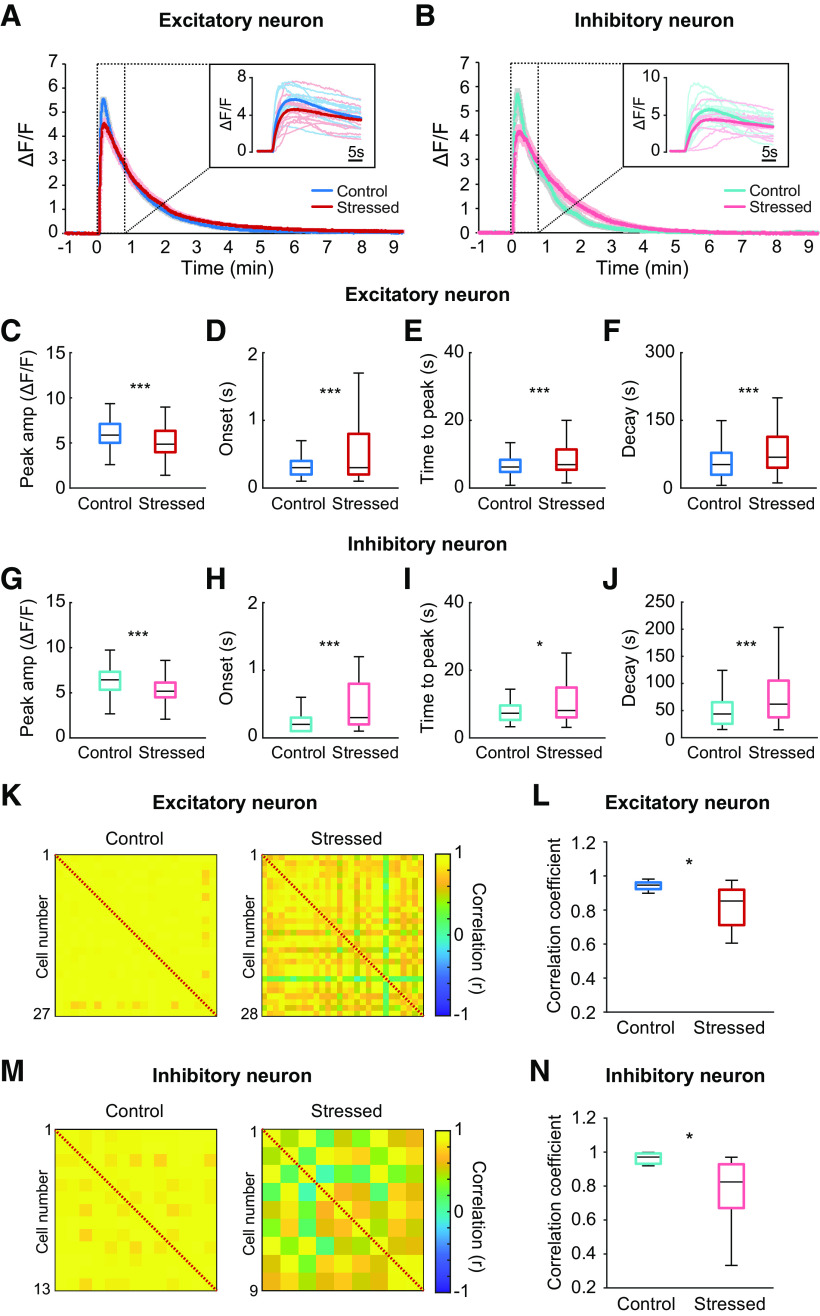
Impairment of calcium transients from both excitatory and inhibitory neurons and neuronal coherence elicited by NMDA simulation under acute stress. ***A***, Average calcium traces of excitatory neurons in response to NMDA stimulation in control and acutely stressed mice. Inset, Individual calcium traces and average calcium traces during the 30 s after the stimulation. ***B***, Average calcium traces of inhibitory neurons in response to NMDA stimulation in control and acutely stressed mice. Inset, Individual calcium traces and average calcium traces during the 30 s after stimulation. ***C–F***, Peak amplitudes, onset times, times to peak, and decay times to half-maximum for calcium transients from excitatory neurons in response to NMDA stimulation in control and stressed mice (control: *n* = 11 slices; stressed: *n* = 12 slices). ***G–J***, Peak amplitudes, onset times, times to peak, and decay times to half-maximum for calcium transients from inhibitory neurons in response to NMDA stimulation in control and stressed mice (control: *n* = 11 slices; stressed: *n* = 12 slices). ***K***, ***M***, Correlation matrices between excitatory neurons and between inhibitory neurons from control (left) and stressed mice (right) during the 1 s following NMDA stimulation. Right, Colors in scale represent correlation values. ***L***, ***N***, Box plots of the mean correlation coefficients of excitatory (***L***) and inhibitory (***N***) neurons from control and stressed mice (control: *n* = 11 slices; stressed: *n* = 12 slices). Box-and-whisker plots represent the data as described in the legend to Figure 2. *p < 0.05; ***p < 0.001; Mann–Whitney *U* test.

In acute brain slices, NMDA stimulation activated both excitatory and inhibitory neurons located within 150-200 μm of the stimulated area. The peak amplitudes of NMDA-evoked calcium transients from excitatory neurons were significantly lower in acutely stressed mice than in controls (control vs stressed: 6.05 ± 0.08 vs 5.15 ± 0.10 ΔF/F, *U*_(360,298)_ = 36 903, *p* < 0.001, Mann–Whitney *U* test; Fig. 4*A*,*C*). Moreover, the onset time and time to peak NMDA-induced calcium transients for excitatory neurons were considerably longer in acutely stressed mice than in control mice (onset: control vs stressed: 0.33 ± 0.01 vs 0.63 ± 0.04 s, *U*_(379,323)_ = 50,642, *p* < 0.001; time to peak: control vs stressed: 6.81 ± 0.17 vs 9.76 ± 0.48 s, *U*_(360,298)_ = 41,807, *p* < 0.001, Mann–Whitney *U* test; Fig. 4*D*,*E*). The decay time to half-maximum for stressed mice was also longer than that for controls (control vs stressed: 49.51 ± 1.80 vs 76.08 ± 3.78 s, *U*_(360,337)_ = 43,836, *p* < 0.001, Mann–Whitney *U* test; Fig. 4*F*). Thus, compared with those in control mice, excitatory neurons in acutely stressed mice showed delayed and broader calcium transients. Similar to excitatory neurons, inhibitory neurons were also altered by acute stress. The peak amplitudes of NMDA-evoked calcium transients from inhibitory neurons were also notably smaller in the stressed group than in the control group (control vs stressed: 6.44 ± 0.20 vs 5.26 ± 0.18 ΔF/F, *t*_(136)_ = 4.337, *p* < 0.001, independent *t* test; Fig. 4*B*,*G*). In addition, the onset time, time to peak NMDA-evoked calcium transient, and half-maximum decay time of NMDA-evoked calcium transients for inhibitory neurons were considerably longer in stressed mice than in controls (onset: control vs stressed: 0.24 ± 0.02 vs 1.18 ± 0.24 s, *U*_(69,72)_ = 1541, *p* < 0.001; time to peak: control vs stressed: 7.48 ± 0.35 vs 12.32 ± 1.19 s, *U*_(65,73)_ = 1815, *p* =0.017; half-maximal decay: control vs stressed: 35.33 ± 2.85 vs 57.71 ± 4.10 s, *U*_(65,73)_ = 1507, *p* < 0.001, Mann–Whitney *U* test; Fig. 4*H–J*). Collectively, these findings indicate that acute stress impairs calcium transients from both excitatory and inhibitory neurons in response to NMDA stimulation.

### Cortical neuronal coherence in acute brain slices following NMDA stimulation is undermined by acute stress

Compared with control conditions, acute stress altered the calcium responses of both excitatory and inhibitory neurons to NMDA and enhanced the variation in calcium traces of excitatory and inhibitory neurons in acute brain slices (Fig. 4*A*,*B*). Thus, we examined whether functional networks between neurons are also affected by acute stress. To determine the coherence of neural activities, we calculated the correlation coefficient for every pair of neurons during the 1 s after the stimulus, when the highest correlated activity was shown in both groups. The correlation values between excitatory neurons were smaller for acutely stressed mice than for controls (control vs stressed: 0.93 ± 0.01 vs 0.89 ± 0.02, *U*_(11,12)_ = 32, *p* = 0.036, Mann–Whitney *U* test; Fig. 4*K*,*L*), implying that neuronal coherence was generally weakened by acute stress. Likewise, acute stress considerably reduced the mean correlation coefficient between inhibitory neurons (control vs stressed: 0.96 ± 0.01 vs 0.89 ± 0.02, *t*_(18.4)_ = 2.651, *p* = 0.016, independent *t* test; Fig. 4*M*,*N*).

**Figure 5.**
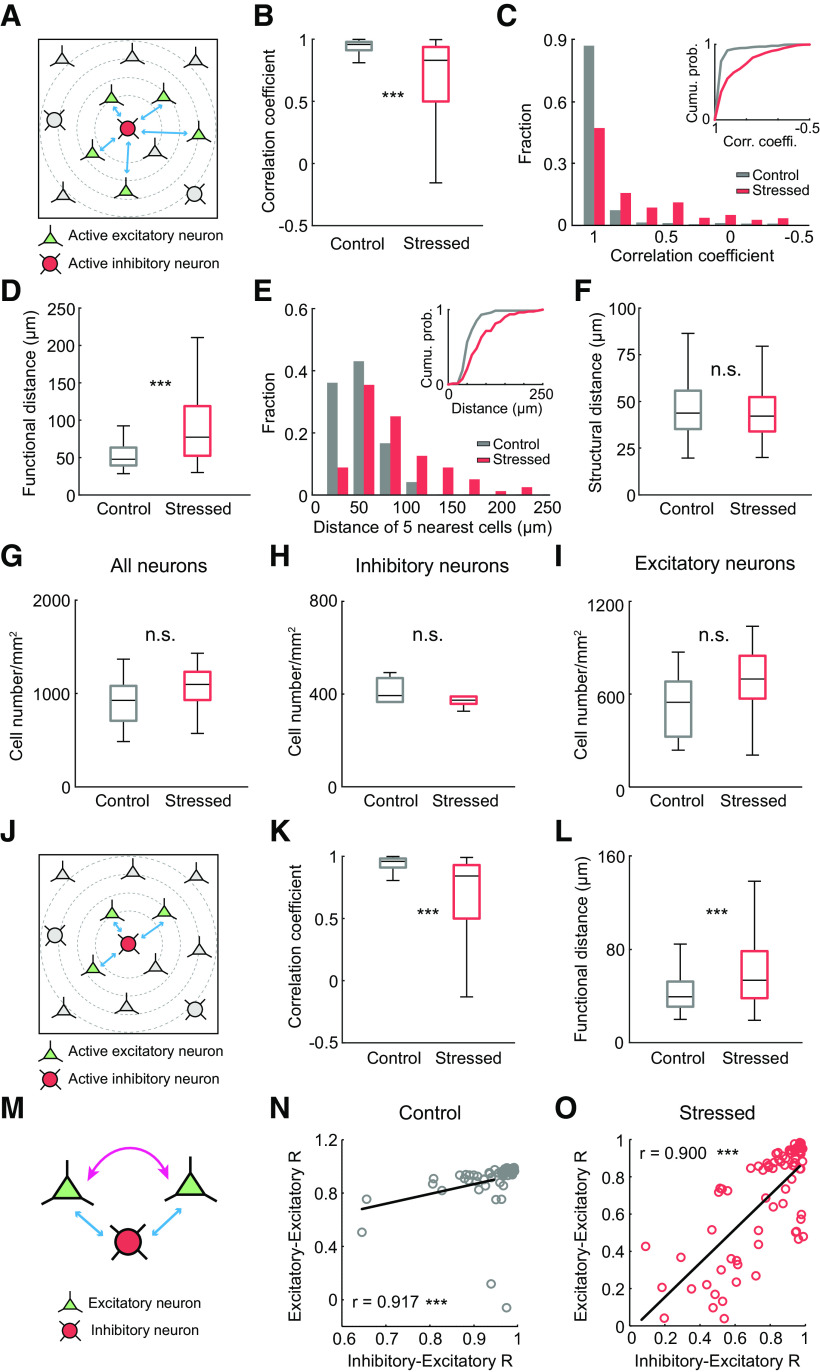
Impairment of neural coherence between excitatory and inhibitory neurons under acute stress. ***A***, Schematics represent the neural connections between a single GABAergic interneuron and the 5 nearest pyramidal cells. Red cell is an active GABAergic neuron. Green cells are the 5 closest active pyramidal neurons to the GABAergic neuron. ***B***, Box plot of the mean correlation coefficients between excitatory and inhibitory neurons (control: *n* = 72 pairs from 11 slices; stressed: *n* = 79 pairs from 12 slices). ***C***, Histogram represents the distribution of the correlation coefficients for control and stressed mice. Inset, A cumulative probability plot of the correlation coefficients. ***D***, Box plot represents the mean functional distances of the 5 nearest pyramidal neurons from a single GABAergic interneuron (control: *n* = 72 pairs from 11 slices; stressed: *n* = 79 pairs from 12 slices). ***E***, Histogram represents the functional distances of the 5 nearest pyramidal neurons from a single GABAergic interneuron in control and stressed mice. Inset, A cumulative probability plot of the mean functional distances. ***F***, Box plot represents the mean structural distance of the 5 nearest pyramidal neurons from a single GABAergic interneuron (control: *n* = 277 pairs from 6 slices; stressed: *n* = 273 pairs from 6 slices). ***G–I***, Box plot represents the numbers of excitatory, inhibitory, and total neurons in control and stressed mice (control: *n* = 6 slices; stressed: *n* = 6 slices). ***J***, Schematic images represent the neural connections between a single GABAergic interneuron and the 3 nearest pyramidal cells. Red cell is a single GABAergic neuron. Green cells are the 3 pyramidal neurons nearest to the GABAergic neuron. ***K***, Box plot of the mean correlation coefficients between excitatory and inhibitory neurons (control: *n* = 72 pairs from 11 slices; stressed: *n* = 79 pairs from 12 slices). ***L***, Box plot represents the mean functional distances of the 3 nearest pyramidal neurons from a single GABAergic interneuron (control: *n* = 72 pairs from 11 slices; stressed: *n* = 79 pairs from 12 slices). ***M***, Schematic images showing the correlations between excitatory neurons and the correlations between excitatory and inhibitory neurons. ***N***, ***O***, Scatter plots represent the linear relationships of correlations between excitatory neurons and correlations between excitatory and inhibitory neurons in control (***N***) and stressed mice (***O***) (control: *n* = 62 pairs from 11 slices; stressed: *n* = 77 pairs from 12 slices). Box-and-whisker plots represent the data as described in the legend to Figure 2. ***p < 0.001; n.s., not significant; Mann–Whitney *U* test and Spearman's test.

Given these results, we investigated whether the neural networks between excitatory and inhibitory neurons are also impaired under acute stress conditions. Since GABAergic interneurons are sparse compared with pyramidal neurons, we investigated the relationship between excitatory and inhibitory neurons based on a single GABAergic neuron and the 5 pyramidal neurons nearest to that GABAergic neuron (Fig. 5*A*). The mean correlation coefficient between excitatory and inhibitory neurons was considerably lower in acutely stressed mice than in control mice (control vs stressed: 0.90 ± 0.01 vs 0.68 ± 0.02, *U*_(360,395)_ = 32,205, *p* < 0.001, Mann–Whitney *U* test; Fig. 5*B*). Compared with the control conditions, acute stress generally weakened neural activity correlations between excitatory and inhibitory neurons (Fig. 5*C*).

Then, we hypothesized that, if neuronal transmission is intact under acute stress, the distance from one activated cell to the other activated cell might be similar in acutely stressed mice as in control mice. Thus, we determined the average distance between a single GABAergic neuron with an increased calcium signal after NMDA stimulation and the 5 pyramidal neurons with increased calcium signal after NMDA stimulation closest to that GABAergic neuron, which was defined as the functional distance in this study. The functional distance between the active GABAergic neuron and the 5 closest active pyramidal neurons was greater under acute stress conditions than under control conditions (control vs stressed: 53.71 ± 2.37 vs 85.83 ± 4.87 μm, *U*_(72,79)_ = 1403.5, *p* < 0.001, Mann–Whitney *U* test; Fig. 5*D*,*E*), implying that there might be deficits in neuronal transmission. To confirm whether acute stress induces cellular loss, we measured the structural distance between a single GABAergic neuron and the 5 closest pyramidal neurons determined by IHC, which showed no difference between the two groups (control vs stressed: 46.29 ± 0.93 vs 44.78 ± 0.94 μm, *U*_(277,273)_ = 35,514, *p* = 0.218, Mann–Whitney *U* test; Fig. 5*F*). Furthermore, we confirmed the number of excitatory and inhibitory neurons in control and stressed mice using IHC (all neurons: control vs stressed: 915.75 ± 127.45 vs 1060.20 ± 119.28 cells/mm^2^, *U*_(6,6)_ = 12, *p* = 0.337; inhibitory neurons: control vs stressed: 379.02 ± 48.35 vs 381.67 ± 19.69 cells/mm^2^, *U*_(6,6)_ = 12.5, *p* = 0.376; excitatory neurons: control vs stressed: 536.73 ± 100.30 vs 678.53 ± 117.99 cells/mm^2^, *U*_(6,6)_ = 14, *p* = 0.522, Mann–Whitney *U* test; Fig. 5*G–I*), which suggests that the weakening of the neuronal coherence was caused not by cellular loss but by impairment of functional cell-to-cell connectivity. Similar findings were also observed when the three nearest pyramidal neurons were used instead of the 5 nearest pyramidal neurons (correlation coefficient: control vs stressed: 0.90 ± 0.01 vs 0.61 ± 0.03, *U*_(216,236)_ = 10,655, *p* < 0.001; functional distance: control vs stressed: 42.77 ± 1.89 vs 63.40 ± 3.89 μm, *U*_(72,79)_ = 1740, *p* < 0.001, Mann–Whitney *U* test; Fig. 5*J–L*).

Since stress reduced functional cell-to-cell connectivity, the correlations between excitatory neurons were compared with the correlations between inhibitory and excitatory neurons to determine the relationship between these correlations. A positive linear relationship was observed between the excitatory neuron-excitatory neuron correlation values and the excitatory neuron-inhibitory neuron correlation values in both control and acutely stressed mice (control: *r* = 0.917, *p* < 0.001; stressed: *r* = 0.9, *p* < 0.001, Spearman's rank correlation coefficient; Fig. 5*M–O*). In contrast, the correlations between inhibitory neurons had a nonsignificant relationship with the correlations between excitatory and inhibitory neurons (data not shown). Interestingly, these findings suggest that the connections between excitatory and inhibitory neurons are tightly coupled with those between excitatory neurons during NMDA-evoked signaling.

### Imbalances in E/I transmission under acute stress conditions

**Figure 6.**
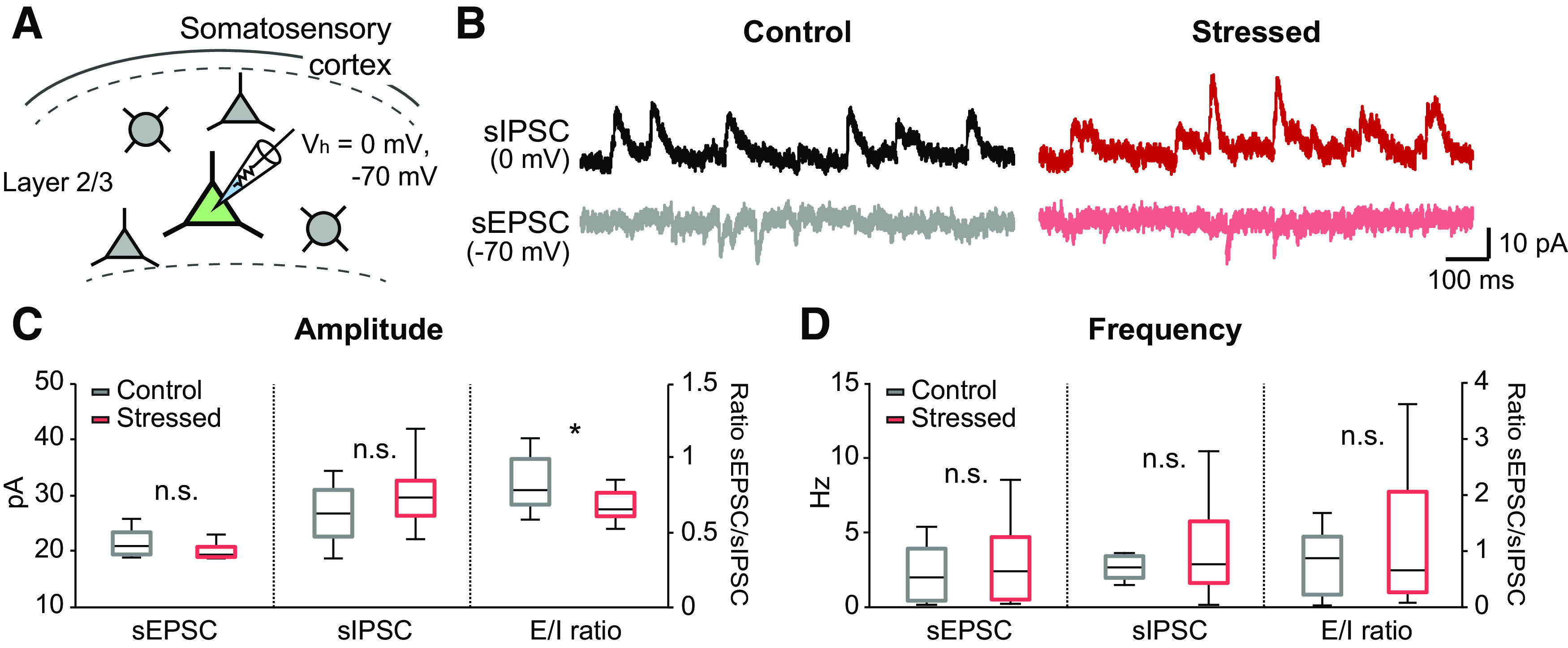
Imbalance of excitatory and inhibitory transmission in acutely stressed mice. ***A***, Schematic showing whole-cell recordings from pyramidal cells in layer 2/3 of the somatosensory cortex. sIPSCs and sEPSCs were recorded from the same neurons at 0 and −70 mV, respectively. ***B***, Representative traces of sEPSCs and sIPSCs in control (left) and stressed mice (right). ***C***, The amplitudes of sEPSCs and sIPSCs and the ratios between sEPSCs and sIPSCs (control: *n* = 12; stressed: *n* = 11). ***D***, The frequency of sEPSCs, sIPSCs, and the ratios between sEPSCs and sIPSCs (control: *n* = 12; stressed: *n* = 11). Box-and-whisker plots represent the data as described in the legend to Figure 2. *p < 0.05; n.s., not significant; Mann–Whitney *U* test and independent sampled *t* test.

Next, we hypothesized that altered excitatory or inhibitory transmission may have caused the reduction in neural activity and the impairment of neural coherence observed in acutely stressed mice. To confirm this hypothesis, we measured sEPSCs and sIPSCs from individual pyramidal neurons located in layer 2/3 of the somatosensory cortex by whole-cell recording (Fig. 6*A*). The amplitude of sEPSCs showed a decreasing trend under acute stressed conditions compared with control condition, although the difference was not significantly different (control vs stressed: 21.42 ± 0.69 vs 20.03 ± 0.43 pA, *U*_(12,11)_ = 40, *p* = 0.110, Mann–Whitney *U* test; Fig. 6*B*,*C*). Moreover, the amplitude of sIPSCs from stressed mice showed an increasing trend compared with those from control mice (control vs stressed: 26.75 ± 1.46 vs 30.10 ± 1.71 pA, *t*_(21)_ = −1.502, *p* = 0.148, independent *t* test; Fig. 6*B*,*C*). Then, we determined the ratio between sEPSC and sIPSC peaks by normalizing the sEPSC peak amplitude to the sIPSC peak amplitude. The results showed that the E/I peak amplitude ratio was significantly decreased by acute stress (control vs stressed: 0.83 ± 0.52 vs 0.68 ± 0.03, *t*_(18.505)_ = 2.337, *p* = 0.031, independent *t* test; Fig. 6*C*), indicating the E/I balance was disrupted under acute stress condition. However, the frequencies of sEPSCs and sIPSCs and the sEPSC/sIPSC frequency ratio were not significantly different between the two groups (control vs stressed: sEPSCs: 3.05 ± 1.05 vs 2.78 ± 0.83 Hz, *U*_(12,11)_ = 62, *p* = 0.805; sIPSCs: 3.38 ± 0.66 vs 4.33 ± 1.22 Hz, *U*_(12,11)_ = 64, *p* = 0.902; E/I ratios: 0.92 ± 0.26 vs 1.11 ± 0.36, *U*_(12,11)_ = 60, *p* = 0.712, Mann–Whitney *U* test; Fig. 6*B*,*D*). Collectively, these findings suggest that the balance of excitatory and inhibitory transmission is disrupted on acute stress.

### Corticosterone signaling via glucocorticoid receptors (GRs) mediates the calcium response and neuronal coherence impairment evoked by NMDA in acute brain slice under acute stress

**Figure 7.**
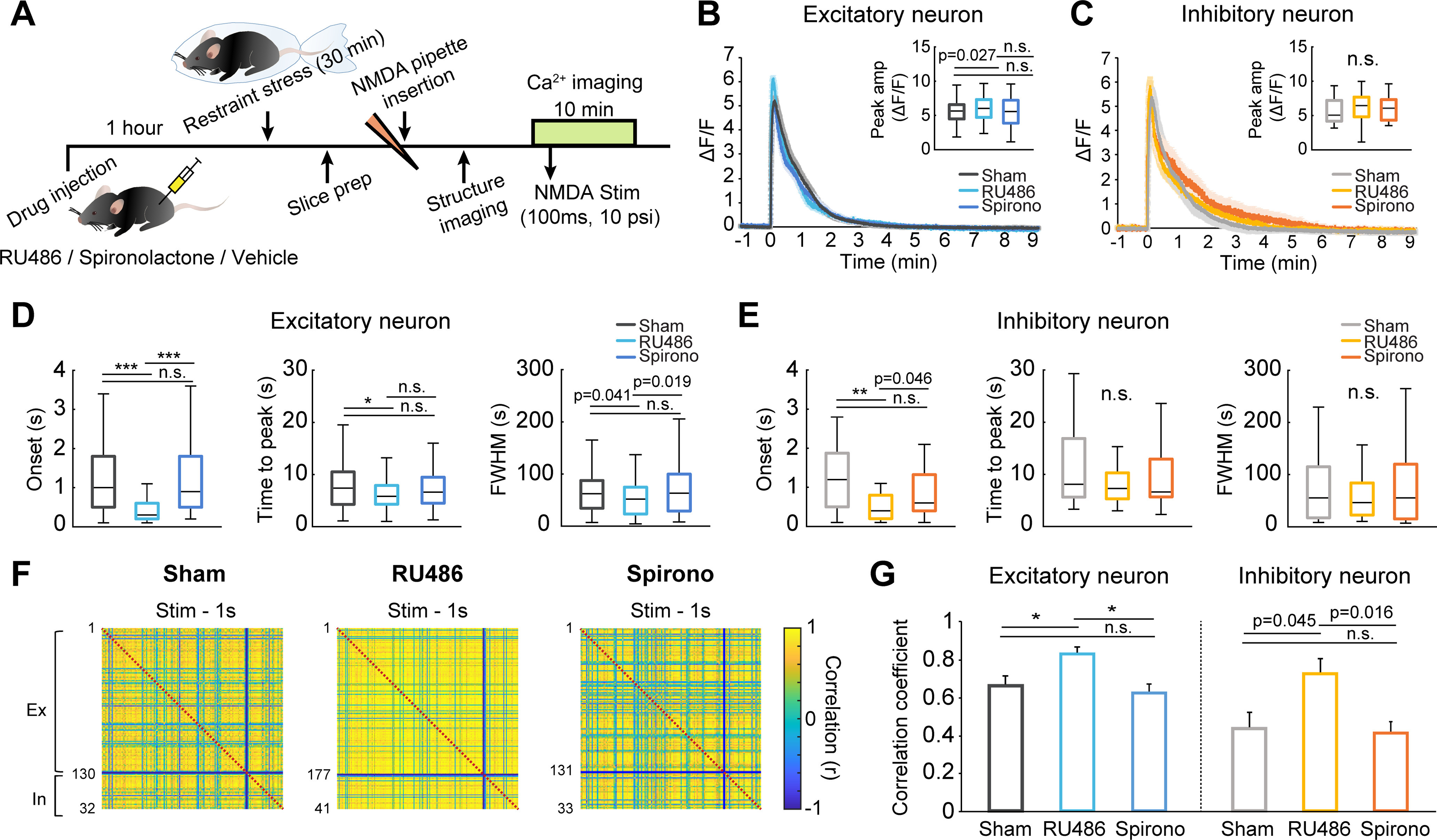
Glucocorticoid signaling via GRs mediates the calcium response and neuronal coherence impairment under acute stress. ***A***, Experimental scheme for *ex vivo* calcium imaging after RU486, spironolactone (Spirono), or vehicle injection. Mice were restrained with plastic bags 1 h after RU486, spironolactone, or vehicle injection. ***B***, Average calcium traces of excitatory neurons in response to NMDA stimulation in sham controls, RU486-treated mice, and spironolactone-treated mice. Inset, Peak amplitudes of calcium signals. ***C***, Average calcium traces of inhibitory neurons in response to NMDA stimulation in sham controls, RU486-treated mice, and spironolactone-treated mice. Inset, Peak amplitudes of calcium signals. ***D***, Onset times, times to peak, and FWHM values of calcium transients from excitatory neurons elicited by NMDA in sham controls, RU486-treated mice, and spironolactone-treated mice (sham: *n* = 129 cells from 6 slices; RU486: *n* = 172 cells from 6 slices; Spirono: *n* = 131 cells from 7 slices). ***E***, Onset times, times to peak, and FWHM values of calcium transients from inhibitory neurons elicited by NMDA in sham controls, RU486-treated mice, and spironolactone-treated mice (sham: *n* = 31 cells from 6 slices; RU486: *n* = 41 cells from 6 slices; Spirono: *n* = 33 cells from 7 slices). ***F***, Correlation matrices of neural responses of excitatory and inhibitory neurons during the 1 s following NMDA stimulation. Right, Colors in scale represent correlation values. ***G***, Bar graphs represent the mean correlation coefficients between excitatory neurons (left) and between inhibitory neurons (right) in sham controls, RU486-treated mice, and spironolactone-treated mice (sham: *n* = 6 slices; RU486: *n* = 6 slices; Spirono: *n* = 7 slices). Box-and-whisker plots represent the data as described in the legend to Figure 2. *p < 0.0167; **p < 0.0033; ***p < 0.00033; n.s., not significant; Bonferroni-corrected. Significant *p* values were determined by Kruskal–Wallis test with Mann–Whitney *post hoc* comparison.

Following exposure to acute stress, increased glucocorticoids interact with two receptors, high-affinity mineralocorticoid receptors (MRs) and low-affinity GRs, to exert biological effects (Reul and Kloet, 1985; Popoli et al., 2011). By acting through MRs and GRs, glucocorticoids have distinct effects on neural activity via genomic and nongenomic action (Prager and Johnson, 2009; Yau and Seckl, 2012). Thus, we examined the effect of blocking signaling of either GRs (with RU486) or MRs (with spironolactone) on neural activity and neuronal coherence following NMDA stimulation in acute brain slices. RU486 (20 mg/kg) or spironolactone (20 mg/kg) was delivered by intraperitoneal injection 1 h before the induction of acute restraint stress (Fig. 7*A*). The peak amplitude of NMDA-evoked calcium transients from excitatory neurons was higher in RU486-treated mice than in vehicle-treated mice (sham vs RU486: 5.54 ± 0.15 vs 6.02 ± 0.14 ΔF/F, *p* = 0.027; Fig. 7*B*). Unlike the RU486-treated mice, the spironolactone-treated mice showed similar peak calcium amplitude as vehicle-treated mice (spironolactone: 5.55 ± 0.20 ΔF/F, *p* = 0.970, χ^2^_(2)_ = 5.748, *p*= 0.056, Kruskal–Wallis test; Fig. 7*B*). Moreover, the onset time and time to peak NMDA-induced calcium transient for excitatory neurons were considerably shorter in RU486-treated mice than in sham controls (sham vs RU486; onset: 1.18 ± 0.09 vs 0.73 ± 0.08 s, *p* < 0.001; time to peak: 8.08 ± 0.42 vs 6.32 ± 0.21 s, *p* = 0.006, Fig. 7*D*). The FWHM for excitatory neurons was smaller in RU486-treated mice than in sham controls (sham vs RU486: 71.22 ± 4.58 vs 53.84 ± 2.26 s, *p* = 0.041; Fig. 7*D*). However, the spironolactone-treated mice and vehicle-treated mice showed no difference in onset time, time to peak, or FWHM of calcium transient from excitatory neurons (spironolactone: onset: 1.42 ± 0.11 s, *p* = 0.210, χ^2^_(2)_ = 77.292, *p* < 0.001; time to peak: 7.89 ± 0.45 s, *p* = 0.456, χ^2^_(2)_ = 7.907, *p* = 0.019; FWHM: 79.42 ± 6.05 s, *p* = 0.664, χ^2^_(2)_ = 6.897, *p* = 0.032, Kruskal–Wallis test; Fig. 7*B*,*D*). Given that no calcium properties were significantly different between RU486-treated mice and control mice following NMDA application (data not shown), RU486 completely reversed the delay and slowing of calcium transients from excitatory neurons observed in acutely stressed mice.

For inhibitory neurons, the onset time of NMDA-evoked calcium transients was notably shorter in RU486-treated mice than in sham controls (sham vs RU486: 1.23 ± 0.14 vs 0.64 ± 0.10 s, *p* < 0.001; Fig. 7*E*). However, the amplitude, time to peak, and FWHM of NMDA-evoked calcium transients from inhibitory neurons in RU486-treated mice were not significantly different from those from inhibitory neurons in sham controls (sham vs RU486: amplitude: 5.68 ± 0.32 vs 6.17 ± 0.34 ΔF/F, *p* = 0.316; time to peak: 10.76 ± 1.21 vs 8.17 ± 0.58 s, *p* =0.277; FWHM: 70.88 ± 10.79 vs 59.70 ± 7.31 s, *p* = 0.811; Fig. 7*C*,*E*). Blocking MRs with spironolactone did not restore the onset time, the amplitude, the time to peak, or the FWHM of calcium transients from inhibitory neurons (spironolactone: onset: 0.86 ± 0.11 s, *p* = 0.086, χ^2^_(2)_ = 11.582, *p* = 0.003; amplitude: 6.01 ± 0.30, *p* = 0.432, χ^2^_(2)_ = 1.361, *p* = 0.506; time to peak: 9.78 ± 1.16 s, *p* = 0.667, χ^2^_(2)_ = 0.913, *p* = 0.633; FWHM: 82.55 ± 14.22 s, *p* = 0.904, χ^2^_(2)_ = 0.148, *p* = 0.929, Kruskal–Wallis test; Fig. 7*C*,*E*). These results suggest that glucocorticoid signaling via GRs mainly alters calcium transients from excitatory neurons in response to NMDA stimulation, subsequently affecting inhibitory neuronal activity.

Following NMDA stimulation, the correlation matrix for RU486-treated mice was a more yellowish color than that for sham controls, implying that RU486 abrogated the neuronal network impairment caused by acute stress (Fig. 7*F*). The mean correlation coefficient for excitatory neurons was significantly higher in RU486-treated mice than in sham mice (sham vs RU486: 0.66 ± 0.04 vs 0.83 ± 0.03, *p* = 0.01; Fig. 7*G*). Moreover, RU486 considerably increased the mean correlation coefficient for inhibitory neurons (sham vs RU486: 0.43 ± 0.08 vs 0.72 ± 0.07, *p* = 0.045; Fig. 7*G*). However, spironolactone did not restore the mean correlation coefficients for excitatory neurons and inhibitory neurons under acute stress conditions (spironolactone: excitatory: 0.61 ± 0.05, *p* = 0.668, χ^2^_(2)_ = 9.520, *p* = 0.009; inhibitory: 0.41 ± 0.06, *p* = 0.715, χ^2^_(2)_ = 6.942, *p* = 0.031, Kruskal–Wallis test; Fig. 7*G*). Together, these results suggest that GR-mediated corticosterone signaling is a key mediator of the calcium response and neuronal network impairment evoked by NMDA stimulation under acute stress.

## Discussion

**Figure 8.**
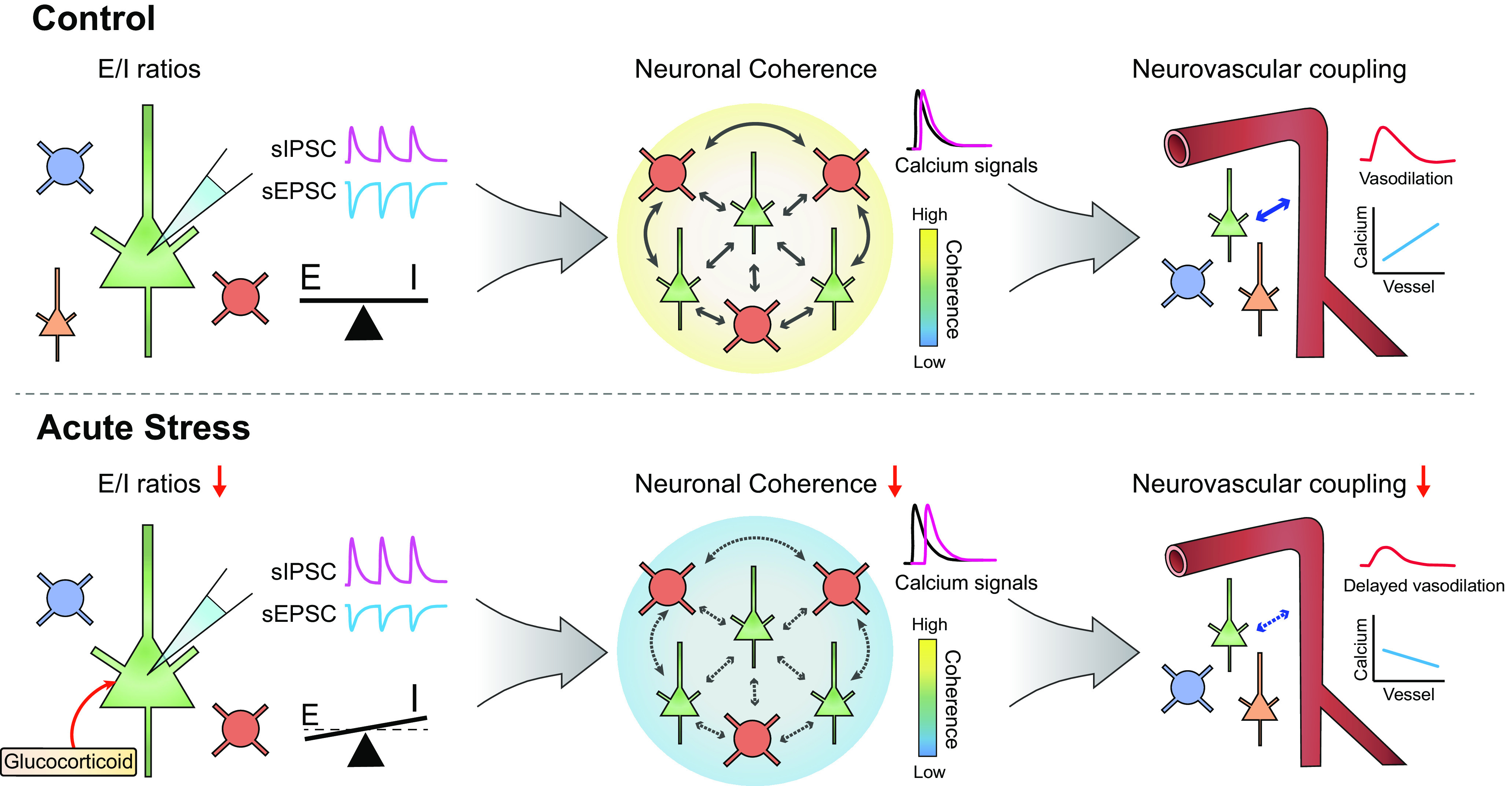
Summary of the relevance of the E/I imbalance, neuronal coherence, and NVC. In control mice, synaptic excitatory and inhibitory transmission is well balanced, inducing highly coherent activity between excitatory and inhibitory neurons. However, increases in glucocorticoid levels induced by a single acute stressor induces a decreasing trend in the amplitude of sEPSCs and an increasing trend in the amplitude of sIPSCs, leading to a reduction in the E/I ratio. Disruption of the E/I balance is accompanied by undermined neuronal coherence, and the positive relationship between the strength of neuronal coherence and the time to peak of the penetrating arteriolar response is completely abolished by acute stress.

In the present study, we demonstrated that acute stress impairs NMDA-evoked vasodilation of penetrating arterioles and calcium transients of excitatory neurons using *in vivo* two-photon imaging. Furthermore, we revealed a close correlation between the strength of excitatory neural coherence and the vascular response, which was shown to deteriorate as neural coherence weakened in acutely stressed mice. To clarify the underlying modulator of excitatory neural coherence, we investigated concurrent calcium signals from excitatory and inhibitory neurons in acute brain slices. Our findings indicated that acute stress impairs coherence not only between excitatory neurons but also between excitatory and inhibitory neurons. Whole-cell recording data revealed that the imbalance of E/I transmission underlies the alteration of neural coherence in acute stressed mice. By blocking GR signaling with RU486, we showed that these neuronal alterations are mediated by GR signaling following an increase in the corticosterone level. In summary, this study demonstrated that, by inducing an E/I imbalance, acute stress contributes to undermining the neuronal coherence between E/I neurons, which ultimately leads to alteration of NVC (Fig. 8).

### Deficits in synaptic E/I transmission under acute stress

Acute stress increases the level of corticosterone and subsequently activates two adrenal steroid receptors, namely, GRs and MRs, both of which induce the alteration of synaptic transmission via nongenomic and genomic pathways (de Kloet and Meijer, 2019). In this study, we found that acute stress decreased the ratio of sEPSCs to sIPSCs with an increasing trend in the sIPSC amplitude and a decreasing trend in the sEPSC amplitude (Fig. 6), implying that there are deficits in the balance between glutamate/GABA transmission under acute stress. In agreement with these results, a previous study reported that the E/I balance in the amygdala is altered by acute stress. Specifically, G. Y. Wang et al. (2016) showed that acute glucocorticoid application induces upregulation of GABAergic neurons and downregulation of glutamatergic neurons in the amygdala, leading to imbalances in interactions between excitatory and inhibitory neurons. However, for decades, accumulating studies have reported that acute stress increases glutamatergic transmission in the mPFC and hippocampus through enhanced glutamate release (Lowy et al., 1993; Musazzi et al., 2010; Satoh and Shimeki, 2010) or alteration of EPSCs (Yuen et al., 2009, 2011). These findings are inconsistent with our results in the somatosensory cortex, possibly because of regional brain differences. Given that our stress model was restrained for only 30 min, these alterations in synaptic transmission might be caused by membrane-associated MRs or membrane-associated GRs, which are well known to rapidly regulate synaptic transmission via a G-protein-coupled pathway (Prager and Johnson, 2009). Membrane-associated MRs enhance presynaptic glutamate release (Karst et al., 2005; Olijslagers et al., 2008), whereas membrane-associated GRs reduce NMDA receptor-mediated currents and calcium conductance while increasing GABA conductance (Di et al., 2003, 2005; He et al., 2003). Thus, the balance between MRs and GRs is important for neuronal transmission. However, the distribution of MR expression is highly heterogeneous, unlike the widespread distribution of GRs (de Kloet et al., 2007), which might lead to regional differences in the ratio between MRs and GRs and, in turn, regional differences in the effects of corticosterone on neuronal transmission. However, further studies are needed to precisely address the exact effects of each types of MR or GR on calcium transients and neuronal coherence from excitatory and inhibitory neurons in somatosensory cortex.

### Influence of an E/I imbalance on neuronal coherence

Since the elaborate synaptic communication between excitatory and inhibitory synapses plays a crucial role in neural networks and normal brain function, an E/I imbalance in synaptic transmission and neural circuits has been implicated in various brain disorders. For example, increased neocortical E/I ratios cause excessive γ oscillations, which are causal factors of autism spectrum disorders (E. Lee et al., 2017). Consistent with these findings, we demonstrated that a disturbed synaptic E/I balance accompanies impaired neural coherence under acute stress. Both excitatory neurons and inhibitory neurons show synchronized activity following NMDA stimulation, which is reflected by neuronal correlations (Cohen and Kohn, 2011). *In vivo* and *ex vivo* experiments revealed that the correlations between excitatory neurons and even between excitatory and inhibitory neurons following NMDA stimulation were significantly reduced in acutely stressed mice (Figs. 2, 5). Since we confirmed that the numbers of excitatory and inhibitory neurons were unchanged in stressed mice (Fig. 5), we concluded that the impairment of neural connections was derived from changes in synaptic transmission in excitatory and inhibitory neurons. Interestingly, the correlations between excitatory and inhibitory neurons exhibited a positive relationship with the correlations between excitatory neurons but not with those between inhibitory neurons (Fig. 5). These findings suggest the possibility that, in the context of NMDA-evoked neural signaling, the coherence between excitatory neurons plays important roles in modulating the coherence between excitatory and inhibitory neurons. Consistent with this idea, a previous study suggested that excitatory signals from excitatory neurons to inhibitory neurons may be responsible for the correlations between excitatory and inhibitory neurons (Hayashi et al., 2018). Collectively, these findings indicate that an E/I imbalance in the acutely stressed brain impairs neural coherence, which may be a contributing factor to the pathophysiology of stress-related diseases.

### Relevance of the E/I balance to the NVC

Accumulating evidence has revealed that precisely coordinated interactions between excitation and inhibition produce γ oscillations (Buzsáki and Wang, 2012), which are known to be strongly positively associated with the hemodynamic response (Niessing et al., 2005; Logothetis, 2008). Our previous study showed that changes in the E/I ratio lead to reductions in γ oscillations accompanied by diminished hemodynamic changes in chronically stressed mice (Han et al., 2019). In this study, we observed coherent activity between excitatory neurons following NMDA stimulation and found that the correlations between excitatory neurons exhibited a strong linear relationship with the vascular reactivity in control mice (Fig. 2). One other interesting finding was that this correlation between excitatory neurons and vascular reactivity was high in both the onset phase and decay phase of the vascular response of penetrating arterioles in control mice (data not shown). However, acutely stressed mice showed a high correlation only in the onset phase of the vascular response, which implies that neuronal coherence is tightly related to not only the onset of the vascular response but also the decay of the vascular response. Why does neuronal coherence have a strong relationship with the vascular response under normal conditions? Prior studies on the relationship between neural activity and BOLD signals have reported that hemodynamic signals are more closely related to local field potentials than spiking activity, implying that the sum of synaptic activity and synchronized activity plays an important role in controlling hemodynamic changes (Arthurs et al., 2000; Lauritzen, 2005). Since cortical oxygen consumption depends on the extracellular ion concentration change following synaptic activity (Mathiesen et al., 1998), synchronized neural activity can increase the timely coherence of oxygen demand, which is reflected by hemodynamic signals, by releasing ions and vasoactive mediators, such as adenosine, prostaglandin E_2_, and NO. Additional evidence has recently shown a direct relationship between the E/I balance and BOLD signals via chemogenetic modulation of excitatory or inhibitory neuronal activity, in which blood flow and functional connectivity changes depending on the E/I ratio. (Markicevic et al., 2020). Together, these findings indicate that changes in the E/I ratio under pathologic conditions can underlie alterations in NVC.

### Limitations and future perspectives

In this study, we used urethane anesthesia for *in vivo* two-photon imaging to avoid the acute stress effects that result from handling and head fixation in experiments on awake animals. Urethane is well known to preserve neurotransmission and NVC (Sceniak and MacIver, 2006; Berwick et al., 2008); and indeed, the *in vivo* data were highly similar to the *ex vivo* data in this study. However, we cannot discard the possibility that the cholinergic tone change in awake state affects NVC (Lecrux et al., 2017); thus, an experiment in awake animals in which stress effects are minimized is required. Second, we only observed links between the neural coherence and penetrating arteriolar response during NMDA-evoked signaling. However, different neural inputs recruit different ratios of excitatory and inhibitory neurons (Lecrux and Hamel, 2016); thus, further study of the relationship between neural networks induced by various synaptic inputs and hemodynamics is required.

In the neocortex, GABAergic interneurons are highly heterogeneous (Rudy et al., 2011), and a previous study reported that pyramidal neurons located in cortical layer 2/3 show an equalized E/I balance that is modulated by parvalbumin-expressing neurons but not somatostatin-expressing neurons (Xue et al., 2014). This study suggests that anatomic and cell type-specific changes in GABAergic interneurons play crucial roles in maintaining the E/I balance. Therefore, it would be very interesting to study the changes in NVC and the E/I balance following cell type-specific modulation of GABAergic interneurons.
